# A Case of Unilateral, Segmental Vascular Lesions: An Unusual Presentation

**DOI:** 10.7759/cureus.44947

**Published:** 2023-09-09

**Authors:** Nayna Nambiar, Adel Iqbal, Emelie E Nelson, Troy A Black, Rashid M Rashid

**Affiliations:** 1 Natural Sciences, Rice University, Houston, USA; 2 Dermatology, John P. and Kathrine G. McGovern Medical School at UTHealth, Houston, USA; 3 Dermatology, Mosaic Dermatology, Houston, USA

**Keywords:** healthy patient, spider angioma, vascular malformations, benign vascular lesions, cherry angioma, hemangioma

## Abstract

We present a case of a diffuse, vascular eruption localized to the left thigh and left abdomen in an otherwise apparently healthy 63-year-old male. The patient reported that the eruption was not bothersome and had been present for as long as he could remember. Due to its benign appearance in nature, the patient declined a biopsy or further follow-up. While reports of diffuse vascular eruptions have been associated with many genetic diseases, this case offers an example of a diffuse vascular eruption in a healthy patient. Further research is needed to understand the potential genetic or environmental factors contributing to the development of such lesions in healthy patients.

## Introduction

Benign vascular lesions refer to non-cancerous growths or abnormalities of blood vessels [1]. Many types of benign vascular lesions have been described, including hemangiomas, cherry angiomas, spider angiomas, and venous lakes [2-4]. It should be noted that the presence of multiple or diffuse vascular lesions has been associated with genetic diseases and vascular anomalies, including Von Hippel-Lindau Syndrome, Sturge-Weber Syndrome, and Parkes-Weber syndrome [5]. In this case report, we present a segmental, vascular eruption occurring unilaterally in an otherwise apparently healthy patient.

## Case presentation

**Figure 1:**
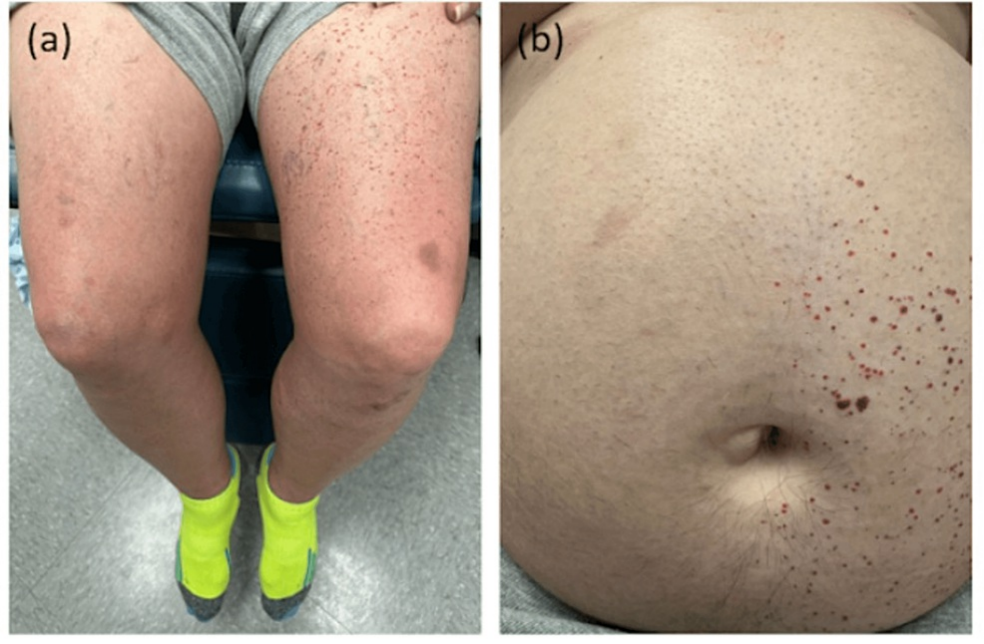
The patient’s left thigh and abdomen upon presentation to the dermatologist.

A 63-year-old healthy male patient presented to dermatology for evaluation of a non-pruritic, non-erythematous, vascular eruption. The patient stated that the eruption was unchanged and that he had had it for as long as he could remember. Notably, the patient reported no significant past medical or dermatologic history, with the exception of the eruption in question. Physical examination of the lesion revealed extensive clusters of vascular lesions in a segmental distribution, affecting the left upper extremity and left abdomen (Fig. 1). Due to the benign appearance of the eruption, the patient declined a biopsy as well as further treatment or follow-up. This case is of particular interest because of its atypical unilateral, segmental presentation as well as its occurrence in an otherwise healthy patient.

## Discussion

Vascular lesions, abnormal growths, or abnormalities of blood vessels can occur on various parts of the body demonstrating both cutaneous and internal involvement. Benign vascular lesions are generally classified into two broad categories: vascular tumors and vascular malformations [1].

Common benign vascular tumors include cherry angiomas, spider angiomas, and venous lakes. Cherry angiomas appear as small, bright red or purple bumps on the skin and tend to increase in number and size with age [2]. In contrast, spider angiomas have a central red macule with small blood vessels radiating outward, resembling spider legs [3]. Finally, venous lakes appear as compressible dark blue or purple macules and are commonly found on elderly patients in sun-exposed areas, such as the lips, ears, or face [4].

Vascular malformations refer to aberrant connections between arteries, veins, and lymphatic vessels [1]. Hemangiomas are one of the most common benign vascular malformations. Usually present at birth, or developing soon after, hemangiomas appear as red, pink, or bluish cutaneous patches or plaques. Hemangiomas tend to grow rapidly during the first few months of life before gradually reducing in size [4].

The diagnosis of benign vascular lesions is most commonly clinical, but histologic evaluation can help distinguish among the various types of vascular lesions. Generally, dermoscopic evaluation of benign vascular lesions characteristically reveals vessels that are expanded or dilated, including red, purple, blue, or black dots, globules, lacunae, and structureless areas, as well as linear, linear irregular, hairpin, comma, and arborizing vessels [6]. Biopsy and histologic evaluation of these lesions often show abnormal blood vessels, endothelial cell proliferation, vascular channels, and vascular markers (CD31, CD34, or GLUT-1) [1]. 

The treatment of vascular lesions depends on type, size, location, symptoms, and individual patient preference. Vascular lesions that are asymptomatic and not cosmetically bothersome may not require treatment but should instead be monitored for potential changes. For benign vascular lesions that are cosmetically bothersome, a variety of topical and oral medications can be trialed, including topical and systemic beta-blockers, topical imiquimod, and corticosteroids [7]. Other treatment options include laser therapy, sclerotherapy, embolization, cryotherapy, and surgical excision [7]. Of note, because our patient’s lesions were not clinically bothersome, he elected to forgo treatment at this time.

Extensive, benign vascular lesions, as seen in our patient, have been observed in the context of specific genetic pathologies. For example, Sturge-Weber syndrome, a neurocutaneous disorder, is typified by vascular malformations resulting in facial port-wine birthmarks on the forehead or eyelid (V1 distribution), and in some cases V2 and V3 distributions [8]. Von Hippel-Lindau disease, an inherited neoplastic condition, is associated with the development of hemangiomas in 50% of patients, with a predilection for the retina. These hemangiomas have the potential to leak serum, resulting in the formation of fibroglial bands that may lead to retinal detachment and vitreous hemorrhage. Consequently, this can result in the emergence of glaucoma and/or irreversible vision impairment [9]. Finally, Parkes-Weber syndrome, a rare congenital disorder, has also been associated with various vascular anomalies, including cutaneous capillary malformations, arteriovenous malformations, and arteriovenous fistulas, often occurring in the legs [10].

Based on physical exam findings, we believe our patient’s presentation is most consistent with unilateral, segmental cherry angiomas, or hemangiomas. Although our patient’s particular pattern of presentation is unique due to the extensive, unilateral, and segmental distribution, cherry angiomas are very common, benign findings in adults. In contrast, hemangiomas are often seen in infancy. These lesions are primarily composed of endothelial cells and pericytes and are characterized by a rapid proliferation phase followed by an involution phase [11]. Because our patient has had this eruption for as long as he can remember, a diagnosis of unilateral, segmental hemangiomas is plausible. Finally, hemangiomatosis, the proliferation of blood vessels, both cutaneously and internally, should be included in the differential diagnosis for this patient [12].

## Conclusions

This case report provides a unique contribution to the literature by documenting a rare occurrence of diffuse, unilateral, benign vascular lesions in an apparently otherwise healthy individual. Although a definitive diagnosis was not formally established, the most likely diagnoses are unilateral, segmental cherry angiomas, or hemangiomas. Our findings underscore the importance of comprehensive evaluation and differential diagnosis in the management of vascular lesions, even in the absence of other health concerns. Continued study is necessary to understand the potential genetic or environmental factors that may contribute to the development of such lesions in healthy individuals.
